# Radiation Monitoring Using Personal Dosimeter Devices in Terms of Long-Term Compliance and Creating a Culture of Safety

**DOI:** 10.7759/cureus.27999

**Published:** 2022-08-14

**Authors:** Fahad Qureshi, Aarya Ramprasad, Bogdan Derylo

**Affiliations:** 1 School of Medicine, University of Missouri - Kansas City, Kansas City, USA; 2 Department of Nephrology, Vascular Access Centers, Chicago, USA

**Keywords:** occupational health, quality improvement, dosimeter usage, radiation monitoring, general radiology

## Abstract

Introduction

Radiation-emitting devices are commonplace in the hospital with their ability to produce imaging for diagnoses, However, they hold a risk for device operators due to radiation exposure. Hospital systems have programs where physicians exposed to radiation are required to wear dosimeters to help record total radiation over time. Dosimetry readings over standardized recommendations can lead to hospital image issues and disciplinary action for physicians. This study aimed to discover the true values recorded on dosimeters with radiation exposure and discuss effective ways to encourage compliance with dosimeter usage.

Methodology

The study was completed over a course of 12 months with physicians from three different hospitals. Selection criteria included physicians considered to be “radiation workers” including those who operate x-ray machines, fluoroscopy units, unsealed and sealed isotopes, or those exposed to other sources of gamma or high-energy beta radiation. Two Plan-Do-Study-Act (PDSA) cycles were implemented. The first cycle was the first six months of the study and the second cycle was the second six months of the study. The first PDSA cycle had planned dosimeter reading check-ins every month. After this cycle ended, physicians were sent a survey anonymously asking if they had ever intentionally left behind their dosimeter. In the second PDSA cycle, a planned policy change was put into action where penalties for physicians who went over the recommended dosage were stopped. A monthly educational meeting where a discussion on the risks of radiation as well as protective mechanisms was implemented instead. The same monthly check-ins for dosimeter reading monitoring were employed again with the same survey regarding dosimeter adherence and usage being sent out at the end of the second cycle. Run charts were created to determine whether the policy change showed statistically significant differences in dosimetry readings.

Results

Protocol changes led to statistically significant (p<0.05) differences in radiation exposure recorded throughout the hospital systems. The primary PDSA cycle readings showed that hospital systems one (n=118), two (n=71), and three (n=32) had readings of 3.90 mSv, 2.55 mSv, and 2.02 mSv, respectively, which were all under the annual recommended dose limit of 10 mSv maximum per six months. However, an average of 94.4% (n=221) of physicians across all hospitals admitted to not using the dosimeter. In the second PDSA cycle after the policy change, the radiation doses were higher with an increase in the average cumulative dose at hospital system one of 255%, 328% at system two, and 323% at system three. Hospital systems one and two were both over the yearly limit of 20.0 mSv (7.70 mSv over for system one and 1.86 mSv over for system two) while system three remained under. The number of physicians who stated they always used the dosimeter during the second PDSA cycle increased to 83.9% in-hospital system one, 90.2% in-hospital system two, and 93.8% in-hospital system three.

Conclusion

Creating a culture of safety is critical for physician compliance. A comfortable work environment without unreasonable consequences creates an environment where physicians can focus on their health and safety while also doing what is in the workplace’s best interest. This culture can best be made with more collaboration between administrative staff and workers to create a trustworthy experience in hospital systems.

## Introduction

Medical innovations continue to provide us with ways to advance patient care by providing quicker diagnoses and more tailored treatment plans. One significant innovation that has grown dramatically in the past few decades is the increase in diagnostic medical imaging. Medical imaging is an essential component of continuity of care as developments in pathological processes can be monitored over long periods with cross-system accessibility present for all physicians to read and interpret disease progression. Imaging helps patients by providing earlier screening, diagnosis, treatment selection, and documentable follow-up. Many studies have been conducted analyzing the patterns of imaging used for patients in past years; one study in a great health program showed 377,048 patients undergoing 4.9 million diagnostic tests from 1997 to 2006 with cross-sectional imaging and computed tomography (CT) imaging use doubling and magnetic resonance imaging (MRI) use tripling [1]. Another retrospective cohort study of 135 million imaging examinations showed an increase of 3.7% in CT use between 2013 and 2016 increasing to 5.2% in older adults and a 1.3% increase between 2007 and 2016 with MRI in adults increasing to 2.2% in older adults [2]. Analysis of previous studies make insight into the progression that has been made in the quantity of use of imaging in the past two decades and how it has changed per patient population.

While patient education on the risks and benefits of imaging has been well emphasized in the healthcare field, a critical problem is the issue of physician radiation exposure and how its long-term risks increase with years of practice due to continued accumulation. Medical imaging is an important source of radiation in the population with its dose-dependent side effects including erythema, hair loss, dermal atrophy, fibrosis, desquamation, dermal necrosis, cataracts, decreases in red blood cell production, and infertility. [3]. One study looking at 232 physicians from 2014 to 2016 showed a poor understanding of radiation risk among physicians, with physicians showing a poor understanding of different imaging modalities and only 26% of the participants being able to correctly identify which modalities expose people to ionizing radiation [4]. Another similar study showed that out of 419 physicians, only 48% of the participants had received training about radiation protection and the negative effects of continued exposure [5]. The risk of radiation exposure is pertinent to all populations, even those outside of the patient populations, and is an ongoing topic of discussion in regard to understanding the amounts of radiation exposure along with how to mitigate the risks associated with higher amounts of exposure.

Many hospital systems have created programs where physicians exposed to radiation wear dosimeters that help record total radiation over a period of time. Each system has its own set criteria on who is required to wear dosimeters and what the regulations are in regard to data collection and further analysis by the administration. The National Council on Radiation Protection (NCRP) limits healthcare-associated occupational exposures to radiation to 5,000 millirems (mrem) per year or 50 millisieverts (mSv) with subsections of 15,000 mrem/150 mSv yearly for the lens of the eye and 50,000 mrem/500 mSv yearly for skin and the extremities. The whole-body dose limit is assumed to be a deep-dose equivalent at a tissue depth of 1 cm while the skin and extremities limit is considered to be a shallow-dose equivalent from an external source of ionizing radiation at a tissue depth of 0.007 centimeters (cm) averaged over an area of 10 cm^2^. The lens dose equivalent is the dose equivalent to the lens of the eye from an external source of ionizing radiation at a tissue depth of 0.3 cm [6]. The International Commission on Radiological Protection (ICRP) limits occupational exposure to 20 mSv per year, averaged over defined periods of 5 years with no single year exceeding 50 mSv [7]. Physicians are commonly confronted if excess exposure is recorded, especially if it poses a risk to the integrity of the institution. For this reason, it has been shown that physicians may intentionally not wear their dosimeters to get falsely low ratings to avoid disciplinary action [8]. One study showed that as much as 50% of physicians do not wear their dosimeters or incorrectly wear them which frequently leads to misleading numbers that can be worsened by a paucity of monitoring within the hospital system [9]. Other physicians adhere to dosimeter usage but in an improper manner, including putting the dosimeters under the lead vest so that it does not receive as much exposure. In our study, we aimed to discover the true values behind dosimetry use in the medical workplace among physicians and discuss a safe and effective way to encourage compliance with the usage of dosimeters among all professionals encountering cumulative radiation exposure.

## Materials and methods

To complete the analysis of dosimeter usage and its effect on the physician population, a study over the course of 12 months was completed. Three separate hospital systems were enrolled in this study. All hospital systems were de-identified with 118 physicians participating in system one, 71 in system two, and 32 in system three. Selection criteria included physicians considered to be “radiation workers.” This included those who operate x-ray machines, fluoroscopy units, unsealed and sealed isotopes, or those exposed to other sources of gamma or high-energy beta radiation [10]. All physicians recruited were those who took these cases within their respective hospital systems. No other radiation workers were included in the study with the goal of focusing primarily on physician radiation exposure. These physicians were informed of the chronology of the project. Physicians were not blinded during this study due to current safety laws requiring physicians to know that dosimeter readings are being recorded and measured per standardized guidelines. The study was framed as a quality improvement project. Two Plan-Do-Study-Act (PDSA) cycles were implemented with cycle one comprising the first six months of the study and cycle two being the second six months of the study. The first PDSA cycle was made of monthly required check-ins to record dosimetry readings. A research team member was assigned to monitor and observe the physicians during this time period to assess dosimeter usage. After the first PDSA cycle, the physicians were sent a survey in an anonymous delivery method with no identifiers asking if they had ever intentionally left behind their dosimeters. All physicians were verified via national provider identifier (NPI) number and were then given a unique de-identified number to prevent the repetition of a single individual more than once as well as to uphold physician privacy during the process.

In the second PDSA cycle of our trial, we enrolled all 221 physicians, with subcategorizations of their respective hospital systems noted, into a planned policy change. This policy change included stopping penalizations for physicians who went over the recommended radiation dosage and instead included a segment in a monthly meeting where discussions and lectures were given regarding topics such as types and sources of radiation, health effects of radiation exposure, treatments for radiation exposure and preventative measures. During this second PDSA cycle, we guaranteed no consequences for anyone who went over radiation limits and kept all physicians anonymous. The same monthly dosimeter reading check-ins as well as the survey asking whether a physician had intentionally left behind their dosimeter was repeated to see how the change in hospital policy affected radiation dose as well as dosimeter use and adherence. The same research team member also monitored dosimeter usage among the physicians in the second PDSA cycle to help assess pattern changes within dosimeter usage witnessed in the work environment to help authenticate physician survey responses. To determine whether this policy change feigned statistically significant improvement, a run chart was created for each hospital system’s dosimetry readings to compare values before and after the policy change.

A new component added to the second PDSA cycle was a separate analysis of physicians' understanding of radiation exposure and how educational presentations may affect these knowledge levels. This was implemented by asking a set of questions at the beginning of the second PDSA cycle where the monthly meeting was initiated to assess physicians' understanding of radiation exposure. Questions were pulled from a question bank created by the team which included the topics that would be spoken about during the monthly discussions and lectures. This included topics related to types of radiation, sources of radiation, health effects of radiation exposure, treatments for radiation exposure, and preventative measures. Each participant’s exam had 10 multiple-choice questions that were pulled from the bank. This test was repeated twice, with one at the three-month mark and one at the six-month mark to evaluate how educational presentations may or may not have improved physician knowledge regarding these topics.

## Results

Our results are significantly different depending on whether it was a component of the first PDSA cycle of the study or the second PDSA cycle. The values analyzed for both components were the same, including average dosimeter radiation reading and survey responses per hospital system.

Dosimetry results over the 12-month period 

During the first PDSA cycle, dosimeters were monitored for each physician in each hospital system monthly. This was associated with a monthly check-in to make sure that the device was functioning properly as well as checking the radiation dose for the month and resetting the monitor. Hospital system one had an average cumulative dose of 3.90 mSv, system two at 2.55 mSv, and system three at 2.02 mSv. Since these numbers are over a span of six months, doubling this value to accommodate the time difference would give us 7.80 mSv for system one, 5.10 mSv for system two, and 4.04 mSv for system three to compare to the recommended annual dose limit. All of these hospital systems in the primary six-month period were well below the annual recommended dose limit of 20 mSv. 

In the second PDSA cycle, dosimetry results were recorded once again for each physician in each hospital system monthly. However, physicians were promised no penalty or repercussions if their dosimetry readings were over the limits set by the hospital and were reminded of the monthly educational meetings that would take precedence in this new system. This cycle was also associated with a monthly check-in to make sure that the device was functioning properly as well as checking the radiation dose for the month and resetting the monitor. The radiation doses for the second PDSA cycle were significantly higher, with an increase in the average cumulative dose at hospital system one of 255%, 328% at hospital system two, and 323% at hospital system three. Hospital system one had a six-month average cumulative dose of 13.9 mSv, system two at 10.9 mSv, and system three at 8.56 mSv. Since these numbers are over a span of six months, multiplying them by two would give us 27.7 mSv for system one, 21.9 mSv for system two, and 17.1 mSv for system three to compare to the recommended annual dose limit. Hospital systems one and two were both over the yearly limit of 20.0 mSv according to the ICRP (7.70 mSv over for hospital system one and 1.86 mSv over for hospital system two) but under the limit of 50.0 mSv according to both the ICRP and NCRP. Hospital system three remained under the limit of 20.0 mSv.

**Figure 1:**
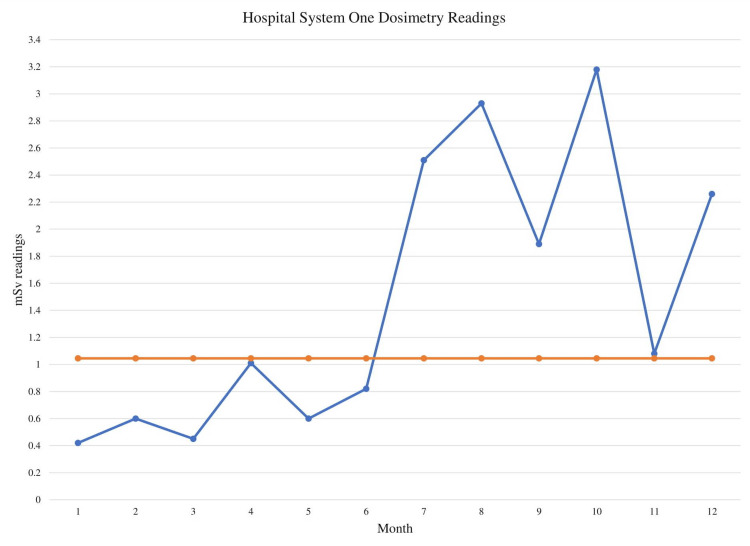
Hospital system one dosimetry readings Blue line: monthly mSv readings; Orange line: median mSv readings

**Figure 2:**
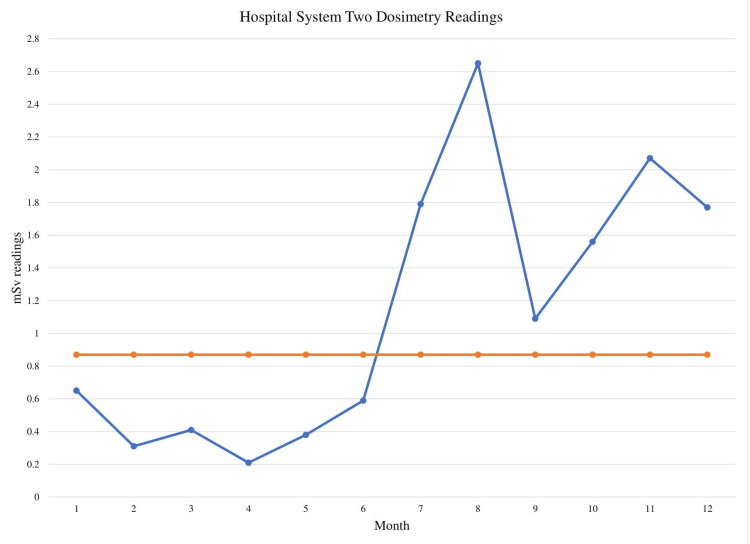
Hospital system two dosimetry readings Blue line: monthly mSv readings; Orange line: median mSv readings

**Figure 3:**
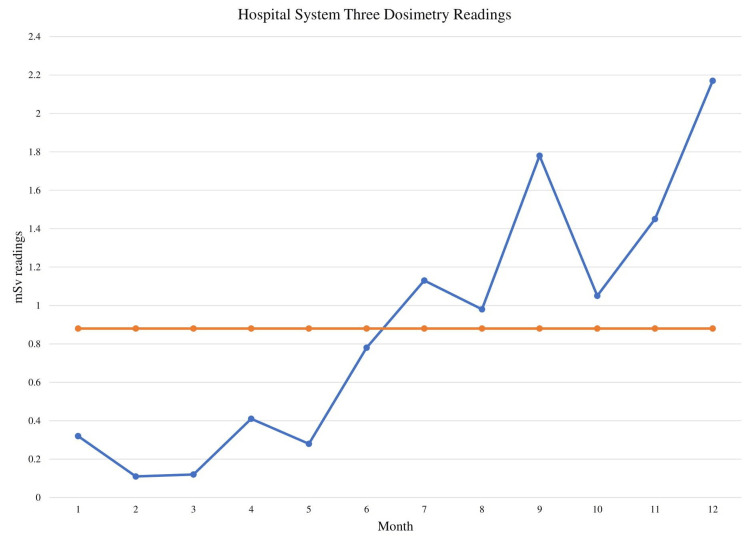
Hospital system three dosimetry readings Blue line: monthly mSv readings; Orange line: median mSv readings

To determine the statistical significance of these changes in value, the mSv dose was graphed on run charts for each individual hospital system to determine statistical significance (Figures 1-3). Analysis showed shifts in the run charts as well as too few runs present for each hospital system. Shifts entail six or more consecutive points all above or below the centerline, which is based on the median value. Shifts with six values each can be seen for every hospital system before and after the planned policy change after month 6 of the study. All three run-charts also had too few runs; runs are considered one or more consecutive data point that is on the same side of the centerline. For a run-chart with 12 data points, a number between three and 10 runs is considered to be a signal of random variation. However, the run-charts for all three hospital systems had two runs each. Both of these interpretations of the run charts are evidence of non-random signals of change meaning there is a less than 5% likelihood that these conditions were met simply by chance [11].

Surveys regarding dosimeter usage before and after the policy change 

After the first PDSA cycle ended with dosimeter monitoring, an anonymous survey was provided to each physician in all three hospital systems to answer on dosimeter usage and adherence. Shockingly, an average of 94.4% (n=221) of physicians across all three hospital systems admitted to not using the dosimeter in the past or during this primary six-month period. Hospital system one had 88.9% (n=118) of physicians not using it consistently while only 11.1% used it consistently. Similar results were seen in both other hospital systems with only 5.70% (n=71) of physicians in hospital system two and 0.00% (n=32) in hospital system three. After the second PDSA cycle ended with dosimetry monitoring, the same anonymous survey was repeated and sent to each physician in all three hospital systems. This survey was slightly modified to ask about adherence in the last six months after the policy change had been implemented. The number of physicians who stated they always used the dosimeter drastically increased, with 83.9% in-hospital system one, 90.2% in-hospital system two, and 93.8% in-hospital system three stating that they consistently used the dosimeter during the second PDSA cycle. While there were still physicians who did not use the dosimeter at least once, this proportion was 16.1% or below between all hospital systems. The results comparing survey results from before and after the policy change are seen in Tables 1-3 for each hospital system. 

**Table 1:** Survey responses for hospital system one

	Before policy change	After policy change
Did not use dosimeter at least once	88.9%	16.1%
Always used dosimeter	11.1%	83.9%

**Table 2:** Survey responses for hospital system two

	Before policy change	Afer policy change
Did not use dosimeter at least once	94.3%	9.85%
Always used dosimeter	5.70%	90.2%

**Table 3:** Survey responses for hospital system three

	Before policy change	After policy change
Did not use dosimeter at least once	100%	6.25%
Always used dosimeter	0.00%	93.8%

Educational presentation effectiveness 

Instead of focusing on administrative repercussions for higher radiation doses recorded by physicians, this study focused on the element of education to see if this could help broaden physician awareness of the dangers and effects of radiation. All physicians from all three hospital systems were grouped into a cohort of 221 participants for examining the test results. Physicians were given monthly briefings during a meeting with education being provided on a topic related to radiation and its exposure. The average test results at the beginning of the second half of the study when no additional education had been provided yet was 49%. At the three-month follow-up mark, it was 61%, and finally, at the six-month mark reaching the end of the study, it was 84% (Table 4).

**Table 4:** Average test results per follow-up month

	n=221 physicians
Pre-test (0 months)	49%
Follow-up test (3 months)	61%
Final test (6 months)	84%

## Discussion

Changes in protocol led to statistically significant differences in radiation exposure in all three hospital systems throughout our study. The primary PDSA cycle showed all three hospital systems to have estimated annual doses far lower than the recommended dose limit of 20 mSv. However, an average of 94.4% (n=221) of physicians across all three hospital systems admitted to either not using the dosimeter, or incorrectly using the dosimeter. Common methods of incorrect use that can help reduce dosimeter readings include forgetting to return the dosimetry badge before leaving work, not monitoring the condition of their badges, wearing the badge under radioprotective gear, and placing it in an improper place, and others [12]. Proper dosimeter use is supposed to be placed on the front of the body in the main area of the torso usually anywhere from the waist to the neck but preferably at the midline [13]. Those who wear lead garments should position the dosimeter outside of any lead protection [14]. These rules vary slightly per institution but all follow the general rules that are outlined by safety commissions that monitor dose regulation [15]. Physicians were found to have incorrect dosimeter usage with speculation regarding whether this is due to intentional manipulation of dosimeter readings or accidental improper technique.

Though only two of the hospital systems went over the limit of an average of 20 mSv per year, the third hospital system had an annual estimate of 17.1 which fared fairly close. Though 50 mSv can be seen as the limit for any year and 20 mSv is recommended per year over a five-year basis, with such high percentage increases in the amount of radiation exposure recorded, this leads to an increased risk of health-related side effects with accumulated dose [16]. The ICRP development of the 20 mSv dose was based on studies that led to the choosing of a dose that correlated to a tolerable consequence (i.e., something that falls short of unacceptable) based on quantifiable attributes of detriment. These attributes included the lifetime probability of death, the reduction of life expectancy, and an increase in the age-specific mortality rate [17]. Some of these consequences can be broken down into somatic effects, genetic effects, and teratogenic effects. Somatic effects include the physician's effects on the body including cancer after years of exposure. Genetic effects can occur in future children and be passed on the future generations. Teratogenic effects can lead to cancer or congenital defects in future children who were exposed to high doses during the embryonic stages of development [18]. All of these risks continue to increase with higher doses of cumulative radiation exposure per year.

Education and training helped physicians within the study to show some improvement in their radiation knowledge base. A 35% increase between the pretest and the final exam was noted among our examinees. Despite this increase in scores, some variability in test scores may be present due to each exam being a random set of questions pulled from the question bank eliminating consistency across each examinee’s test. Tests were given random questions for both pre-tests and final exams to ensure that test-takers did not answer questions based on the memory of the correct answer and instead based it off learned knowledge. Our test score breakdown also was collectively analyzed. This created another weakness as there was no breakdown of how specific types of physicians may or may not have improved their knowledge base during the second PDSA cycle. This information could provide more insight into which types of physicians who are considered radiation workers may have more or less knowledge of the topics surrounding radiation. Improvements in our test scores could be further improved by continued medical education over-scheduled periods of time. This can be facilitated by departments such as occupational safety or other radiation safety departments in a health system. Though many physicians did not score well on the exams, many appeared motivated to learn during our six-month time period. Reputations of workplaces can lead to disregard for safe environments due to fear of losing jobs, reduced pay or being scrutinized during practice. Another issue that seems present is the repetition of the same thing on a daily basis leads to a mental reduction of importance and concern for side effects. Constant exposure to radiation is a part of many of these physicians' lives, and its continued existence in the background can lead to less significance placed on it. This process of desensitization can make the side effects, though generally known to most of these physicians, less jarring and less of a concern on a day-to-day basis, especially with its repercussions not being seen immediately [19]. We propose that this can be counteracted with scheduled education on the topic that serves as a reminder, especially regarding the side effects of long-term radiation therapy, which may reinforce pertinent information.

A strength of our study included the fact that proceedings were done with anonymity while also using research staff to confirm the accuracy of the surveys. This allowed for the most honest results in our surveys due to participants not being in fear of repercussions. Lack of judgment may lead to a more comfortable work environment where physicians are more comfortable sharing their readings truthfully or bringing up concerns regarding monitoring [20]. This allowed our study to provide more honest results that showed the intention behind each decision made by the workers. Limitations of our study include the study being completed with a smaller sample size with increased hospital coverage across the country allowing for more significance to our results. These results cannot be generalized outside of the United States due to systemic differences present in international healthcare. A component that was not further investigated in this study was a breakdown of the different types of physicians who were considered radiation workers. Data were not collected on the specialties for the physicians selected and further analysis in the future with this information could provide insight into what kinds of physicians that encounter radiation have higher or lower rates of exposure. Differences in baseline education regarding radiation versus after further education and examination could also be compared between subspecialties which would allow for a more focused effort on working with physicians who are at higher risk. Our study implemented one idea of how hospital systems can help understand true radiation exposure values in their departments but other possibilities exist as well. Staff who comply with the dosimeter regulations, especially in an environment without fear of certain repercussions, can receive feedback about where and when they are receiving higher radiation doses, which can help audit behaviors and promote increased safety awareness. In other studies with dosimetry readings placed both outside and inside the lead apron can provide comparisons to be analyzed by the facilities radiation safety department which can take according to action and changes to help reduce doses and educate on how protection can reduce dose exposure. Continued analysis within hospital systems can allow for more accurate breakdowns of radiation dose numbers that are supported by physicians within the system allowing for a collective effort toward lower radiation dose exposure within the hospital system.

## Conclusions

Creating a culture of safety is critical for physician compliance. This culture can only be created via collaboration between staff being monitored and those in charge of the monitoring. Teamwork and understanding of both sides can provide more insight into why some physicians may be more inclined toward not being compliant with dosimetry usage due to the repercussions that exist within their hospital system. Education and training also play a significant part in regards to being a tool for encouraging the enforcement of ideals that should be hospital-wide and engrained in physicians' minds. This includes an understanding of proper dosimetry usage and the side effects of increased radiation doses in the long term. Reminders on a scheduled basis can help reinforce these ideas and help with alternatives to more serious repercussions such as job loss or scrutiny that many physicians fear leading to more misuse of dosimeters. Our study shows that if physicians fear retaliation and penalties, they will not focus on safety regardless of its importance. However, if safety is the goal without unreasonable consequences, they will do what is in their own and their workplace’s best interest.
